# An oriented service multilayer architecture for virtual microscopy in mobile devices

**DOI:** 10.1186/1746-1596-8-S1-S31

**Published:** 2013-09-30

**Authors:** Luis Martínez, Germán Corredor, Marcela Iregui, Eduardo Romero

**Affiliations:** 1 Department of System and Industrial Engineering, Faculty of Engineering, Universidad Nacional de Colombia, Bogotá, Colombia; 2 Multimedia Engineering Program, Faculty of Engineering, Universidad Militar Nueva Granada, Bogotá, Colombia; 3 Departament of Diagnostic Images, Faculty of Medicine, Universidad Nacional de Colombia, Bogotá, Colombia

## Background

The Virtual Slide (VS) is the constructed tool for interaction with a large amount of visual information, using for doing so devices designed to display and interact with the VS, i.e., search of Regions of Interest (RoIs), labeling specific RoIs in the VS, automatic VS or retrieval of certain RoIs [1]. Two main VS advantages, over a glass slide, are the information access and the data maintenance. Disadvantages are related with the computational cost [2]. Overall, slide storing and interaction is carried out from the same device used for display. Mobile devices are of course an extreme case of poor resources [3] and therefore clever navigation strategies are necessary to optimally interact with the VS.

Related to interaction with VS from mobile devices, there are two main issues to be addressed. The former is related to the storage and access to a large quantity of data, the latter concerns the reconstruction and display of the visual information. Several works have used the JPEG2000 compression standard to address the storage needs [4][5,6]. JPEG2000 is an image compression standard designed by the Joint Photographic Expert Group, based on the Discrete Wavelet Transform and the EBCOT encoder [7]. This standard provides several advantages, among others, compression efficiency, lossy and lossless compression and multidimensional data access, i.e., random and multiple resolution data representation and data organization in several quality layers [8]. Likewise, the granularity provided by the standard allows the retrieval of individual packets, guaranteeing transmission of just the number of bytes required to reconstruct particular regions of an image, instead of the whole slide [9].

As it was mentioned, a reconstruction task can be achieved by taking advantage of the granularity in the JPEG2000 standard. However, the data syntax described in the standard rules out the interactive construction of a valid data stream from arbitrarily ordered packets [10]. Moreover, reconstruction and display of the VS is still an open problem because of the high consumption of computational resource when decompressing the bitstream. A well designed architecture must therefore address the reconstruction task under the perspective of an optimal adaptation of the process policies to the problem.

## Material and methods

### Experimental setup

The proposed architecture was evaluated with a virtual slide of 36000x9200 pixels, each pixel corresponding to 0.67 µm^2^. The original Virtual Slide had a size of 995 MB, and after JPEG2000 compression, of 226 MB. To run the storage layer, a distributed file system was deployed using 5 Linux nodes. The machines that form the network have limited processing capacity and low speed hard disks (1 GB of memory, processor of 2.2 GHz and disk of 7200 rpm). To run the data access and the proxy layers, two servers were selected; each with operating system OpenSUSE 11.4, 2.8 GHz 4-core processor and 5 GB RAM. Likewise, to run the Samsung Galaxy Tab 10.1 client, under an operating Android system 3.2, it was selected a device with a 1280×800 display size, 1 GB RAM memory and 1 GHz dual-core Nvidia Tegra 2 processor.

### Architecture overview

**Figure 1:**
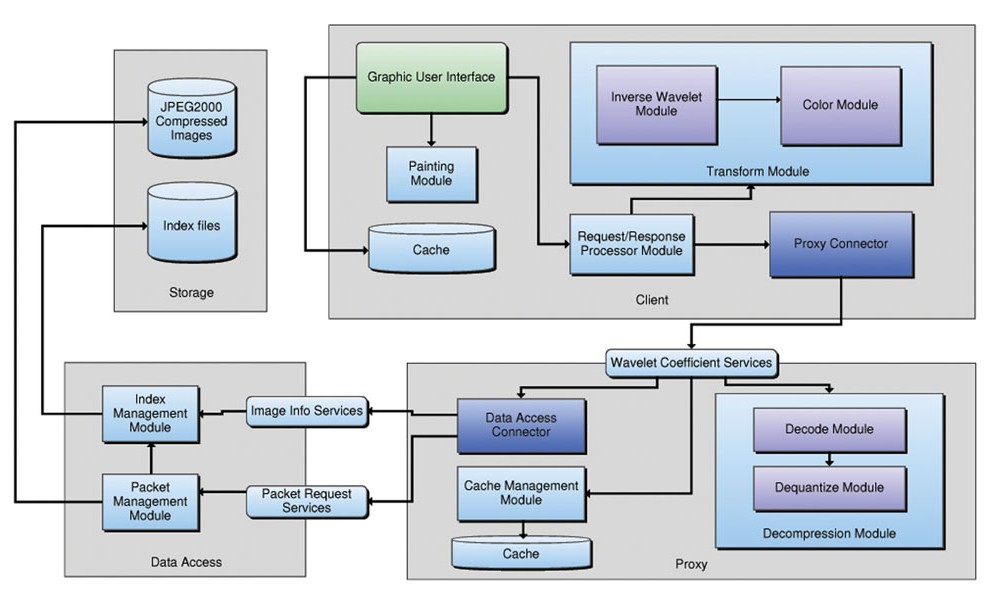
**Multilayer architecture distribution** The figure shows the distribution of the main components within different layers in the proposed architecture.

The proposed architecture exploits the JPEG2000 granularity by dividing the main tasks of the data processing into four layers. The architecture and their main components are shown in Figure 1.

The data storage layer is charged of managing the compressed images. A JPEG2000 compressed image typically contains numerous embedded subsets, each standing for any of a large number of different spatial resolutions, image quality layers and spatial regions. This multidimensional access to data is defined in the JPEG2000 standard as spatially adjacent code-blocks, known as *precincts*. Each precinct is represented as a collection of *packets*, with one packet per quality layer, resolution level and component [10]. The logical structure of this compressed file is stored in an index file, along with the compressed image, and is herein used to navigate through the compressed file. The second, the data access layer, provides the required services to interact with the information stored in the previous layer. A loosely coupled architecture is maintained by providing the required services to interact with the minimal unit of information, i.e. services to retrieve specific image packets and services to retrieve the compressed image header. The services provided by this layer handle each request independently, thereby guaranteeing a simultaneous information access.

The proxy layer is the backbone of the interaction with the data stored in the first layer. This layer is responsible for two important tasks. The first task is to facilitate interaction with data retrieved by the data access layer, and the second one consists in providing efficient access to the previously requested packets. Provided that the present architecture is service oriented, this third layer receives and sends messages, from which the raw data must be extracted. For doing so, this layer has a decompression module, containing the functions to manage and to map the incoming messages from the data access layer. In addition, this layer implements a simple cache module, charged of checking and/or requesting the required packets to fulfill a requested region. Also, a communication API was designed for mobile devices because of the communication problems presented when using conventional web services since they are difficult to process in such limited devices.

Finally, the client layer is a standalone prototype, whose main function is to map the requested regions to list of packets, and reconstruct Virtual Slides, using information provided by the previous layers. The client uses the communication API and retrieves the required information for reconstruction. The client layer uses also a transformation module that allows the final display of inverse transformed wavelet coefficients.

## Results and discussion

The proposed architecture was twice tested. Firstly, it was requested a variable region size, with constant resolution and quality values. Secondly, the requested regions were refined by requesting higher quality layers. Results show the advantage of retrieving and decompressing (decoding) a particular image region instead of the whole slide. In the first test, the time for resolving a requested region is proportional to the number of required packets to reconstruct it. These results are presented in the Table 1.

**Table 1:** Requests with different region size

Window size	Transmission time (ms)	Reconstruction time (ms)
1024x1024	34.9±5.65	162.6±4.7

2048x2048	79.6±6.64	244.1±1.1

4096x4096	471±68.16	613.7±36.79

8192x8192	1421±180.38	1881.8±46.83

The table shows the evolution of the transmission and reconstruction times while the client requests different region sizes of a virtual slide.

In the second test, the time between the requested layers is relatively small, probably because most of the relevant information is mainly compressed in the first layers, leaving small refinement details for the last ones. These results are presented in the Table 2.

**Table 2:** Requests with refinement process

Quality layer	Transmission time (ms)	Reconstruction time (ms)
1	83.1±25.39	245.9±5.49

2	94.4±25.55	249.7±1.16

3	97.3±25.66	250.3±1.57

4	99.5±24.13	250.5±0.53

5	103.4±25.07	254.9±6.66

6	99.8±7.22	258.2±8.57

7	108.9±24.85	268.9±27.09

8	124.5±51.03	265.5±28.48

The table shows the evolution of the transmission and reconstruction times while the client requests different layers for the same region of a virtual slide.

## Conclusions

In this article, it was presented a distributed multi-layer architecture that supports interaction between its layers through a service-oriented scheme. It was shown that retrieval and reconstruction times are relatively slow using a refinement process by quality layers.

## List of abbreviations

API: Application Programming Interface; ROI: Region of Interest; VM: Virtual Microscope; VS: Virtual Slide

## Competing interests

The authors declare that they have no competing interests.

## Authors' contributions

LM implemented, deployed, and evaluated the web services for each layer and the results obtained in the reconstruction process. GC implemented and tested the client prototype, the communication API and the codestream transmission for Android Operating System. MI proposed, implemented and tested the index design, construction and parsing, and developed the fundamental ideas underlying this architecture. ER conceived the study, developed the fundamental ideas underlying this architecture, participated in the experimental design and was the director of the whole project. All authors read and approved the final manuscript.
